# The Effect of Alarm Fatigue on the Tendency to Make Medical Errors in Surgical Intensive Care Nurses: A Correlational Study Examining the Role of Moderating Factors

**DOI:** 10.3390/healthcare13060631

**Published:** 2025-03-14

**Authors:** Muaz Gülşen, Sevban Arslan

**Affiliations:** Surgical Nursing Department, Faculty of Health Sciences, Çukurova University, 01330 Adana, Turkey; sarslan@cu.edu.tr

**Keywords:** alarm fatigue, medical error, surgical intensive care unit, nurse

## Abstract

**Introduction:** In surgical intensive care units, monitoring and interventions are performed utilizing advanced technologies. The warning alarms of these devices jeopardize patient safety by inducing fatigue in staff. **Aim:** The objective is to assess the impact of alarm fatigue on the tendency for medical errors among nurses in surgical intensive care units. **Method:** The current study employed a cross-sectional and correlational design. Data were gathered from 201 surgical intensive care nurses through an online survey approach and snowball sampling technique. The data collection instruments employed were the “Personal Information Form”, the “Alarm Fatigue Scale”, and the “Medical Error Tendency Scale in Nursing”. **Results:** The mean total score for alarm fatigue among nurses in surgical intensive care units was found to be 16.42 ± 5.47, while the mean total score for the tendency to make medical errors was 180.57 ± 24.32. A negative moderate correlation was identified between alarm fatigue and a tendency for medical errors. This finding indicates that as alarm fatigue increases, the score reflecting the tendency to make medical errors decreases; however, this decrease suggests an actual increase in the tendency for medical errors. Nurses’ alarm fatigue accounted for 14.5% of the total variance in the tendency to commit medical errors. A unit increase in alarm fatigue was found to correlate with a 0.381 unit increase in the likelihood of medical errors. **Conclusions:** Nurses exhibited moderate levels of alarm fatigue and a tendency for medical mistakes. The tendency for medical errors escalated markedly with the rise in alarm fatigue.

## 1. Introduction

Alarm fatigue is a condition that occurs when healthcare professionals are exposed to repeated alarms, leading to a decrease in the individual’s sensitivity to alarm sounds and a diminished ability to distinguish real threats [1]. This situation results in significant safety risks, particularly in surgical intensive care units, where critical patient monitoring is crucial. Alarm fatigue is not just an individual issue, but also a systemic vulnerability [2]. A study revealed that nurses responded multiple times to 30% of alarms from patients in intensive care, with 57.7% of nurses experiencing desensitization to these alarms over time [3]. Jeong and Kim (2023) and Ding et al. (2023) observed that nurses in intensive care suffered moderate alarm fatigue, resulting in depersonalization and burnout [4,5].

Desensitization and burnout resulting from alarm fatigue manifest in behaviors such as silencing alerts, reducing volume, postponing responses under the assumption that the signal is erroneous, or removing the alarm without assessing the patient [6,7,8]. These behaviors affecting patient care and safety may lead to the death of patients [8]. The Joint Commission of the American Association of Intensive Care Nurses identifies alarm fatigue as a significant issue jeopardizing patient safety and personnel well-being in the Alarm Management Package Application released in 2022 [9]. The American Emergency Care Research Institute’s 2023 study on the “Top Ten Health Technology Hazards” underscores the paramount need for alarms, warnings, and notifications for the protection of patients and staff, asserting that this matter should be prioritized [10]. The sixth aim of the National Hospital Safety Goals, announced by the United States Joint Commission in January 2024, is to “Reduce harm in clinical alarm systems” [11]. Moreover, alarm fatigue has been recognized as one of the 10 principal health technology hazards in the Emergency Treatment Research Institute’s 2020 study [1]. Consequently, numerous countries and organizations acknowledge alarm fatigue as a significant issue at both national and international levels [9,10,11]. Despite this awareness, in the literature, studies on factors influencing alarm fatigue [6,12,13] primarily focus on its effects on stress [2], mental workload [1,14], and performance [15,16]. However, there is a lack of studies examining the relationship between alarm fatigue and medical error tendency [17]. While the current research includes nurses working in all intensive care units, it should be noted that each unit has different physical conditions. Specifically, there is a need for a study focused on surgical intensive care units, where emergency interventions are frequent and the diversity of medical devices is high. It is crucial to investigate the effects of alarm fatigue in these units in detail, especially in such critical and complex environments.

### 1.1. Aim of the Study

The aim of this study is to determine the effect of alarm fatigue on the tendency to make medical errors in nurses working in surgical intensive care units and to investigate the possible moderating effect of nurses’ demographic and occupational characteristics on this relationship.

### 1.2. Study Questions

What are the levels of alarm fatigue and medical error tendency in nurses working in surgical intensive care units?

How are the demographic and occupational characteristics of nurses working in surgical intensive care units related to alarm fatigue and medical error tendency?

Is there a significant relationship between alarm fatigue and medical error tendency?

Do the demographic and occupational characteristics of nurses act as a moderating factor in the relationship between alarm fatigue and medical error tendency?

## 2. Method

### 2.1. Study Design

This study is structured within the framework of Donabedian’s Quality Model, and conducted with a cross-sectional and correlational design based on the Structure–Process–Outcome components. In the context of the model, alarm fatigue is considered a “process” variable, while the tendency to make medical errors is evaluated as an “outcome” variable. The demographic and professional characteristics of nurses are examined as regulatory (moderator) variables that may alter this relationship. This theoretical framework allows for a more comprehensive analysis of the relationship between alarm fatigue and the tendency to make medical errors, aiming to provide significant findings in terms of patient safety and quality management in healthcare services. Additionally, this study is reported in accordance with the STROBE (Strengthening the Reporting of Observational Studies in Epidemiology) guidelines for cross-sectional studies [18].

### 2.2. Place and Date of the Study

This research was conducted at six public hospitals located in Adana province, Turkey, from August 2023 to June 2024.

### 2.3. Population and Sample

The population of this study consists of a total of 412 nurses working in the surgical intensive care units of six public hospitals in Adana, Turkey. The sample size was determined using the known population sample size formula (n = N t^2^ pq/d^2^ (N − 1) + t^2^ pq), with t = 1.96 and d = 0.05, as suggested by Salant and Dillman (1994) [19]. Based on this calculation, it was determined that at least 198 nurses should be included in this study, with a 95% confidence interval and a ±5% margin of error.

In the data collection process, in order to reach the target group more effectively, the electronic link to the survey and information text about the research were shared on social media platforms commonly used by the nurses. To increase the sample size and strengthen the representativeness, the snowball sampling method was used. In this method, participants were asked to forward the survey link to other surgical intensive care nurses they knew. As a result of the strategies applied, the minimum sample size was exceeded, and the representativeness was improved. A total of 201 nurses participated in this study, reflecting the diversity among nurses working in surgical intensive care units and providing a reliable basis for the generalizability of the data.

During the data collection process, various measures were taken to reduce nonresponse bias. After the survey link was shared on social media platforms, participants were reminded of the importance of completing the survey through informational text displayed when they first clicked on the link. This informational text also stated that the survey was conducted anonymously, and therefore no direct or personal reminders would be sent. Assurance was given that the anonymity of the participants would be maintained, thus minimizing confidentiality concerns. These methods helped reach a wider participant base and reduce the nonresponse rate. Additionally, to reduce common method bias, the survey was conducted anonymously, and some reverse-coded items were used to minimize social desirability bias in the responses.

### 2.4. Data Collection Tools

A “Personal Information Form [7,12,17]”, the “Alarm Fatigue Scale [20,21]”, and the “Medical Error Tendency Scale in Nursing [22]” were used to collect the data.

**Personal Information Form:** The variables in this form were established by the researchers in alignment with the objectives, research questions, and target demographic of this study, and substantiated by existing literature [7,12,17]. Consequently, the objective was to enhance the accuracy and dependability of this study. The form includes nine aspects that inquire about the nurses’ age, gender, marital status, educational level, length of employment in the profession, experience in the critical care unit, tenure in intensive care, work style, and weekly working hours.

**Alarm Fatigue Scale:** This scale, developed by Torabizadeh et al. in 2017 [20], was adapted to Turkish, and its validity and reliability were examined by Kahraman in 2020 [21]. The scale comprises nine items and utilizes a 5-point Likert-type scoring system. The overall scale score can range from 0 to 36. The scoring system categorizes alarm fatigue levels as follows: 0–12 signifies a “low level”, 13–24 denotes a “moderate level”, and 25–36 represents a “high level” of alarm fatigue. A rise in the score suggests that alarm fatigue adversely impacts nurse performance. The scale’s Cronbach’s alpha coefficient was calculated to be 0.80 [21]. This study calculated a value of 0.92.

**Medical Error Tendency Scale in Nursing:** Developed by Özata and Altunkan (2010), this scale uses a five-point Likert-type rating system [22]. The scale consists of five subscales: medication and transfusion practices, hospital infections, patient monitoring and material safety, communication, and falls. Higher scores on the scale indicate a lower tendency to make medical errors. Based on this, a score range of 49–114 is considered high, 115–180 is considered moderate, and 181–245 is considered low tendency to make medical errors.

The scale’s validity and reliability have been evaluated through comprehensive analyses. For content validity, expert opinions were consulted, and the content validity ratio was calculated. In terms of construct validity, the Kaiser–Meyer–Olkin sample adequacy measure was found to be 0.899, and Bartlett’s Test of Sphericity yielded a value of 8.954 (*p* < 0.01), indicating that the data were suitable for factor analysis. As a result of the factor analysis, a structure consisting of 49 items and 5 factors, which explained 52.149% of the total variance, was obtained, with factor loadings ranging from 0.268 to 0.721 [22].

For the reliability analysis, item-total correlations were found to be above 0.25. The Cronbach’s alpha internal consistency coefficients for the subscales were as follows: 0.96 for medication and transfusion practices, 0.93 for hospital infections, 0.89 for patient monitoring and material safety, 0.85 for communication, and 0.88 for falls. The overall Cronbach’s alpha coefficient for the scale was 0.954, indicating high reliability. This high reliability coefficient demonstrates that the scale has strong internal consistency and is a reliable measurement tool for evaluating nurses’ tendency to make medical errors [22]. In this study, Cronbach’s alpha internal consistency coefficients for the subscales were calculated as follows: 0.72 for medication and transfusion practices, 0.75 for hospital infections, 0.75 for patient monitoring and material safety, 0.80 for communication, and 0.80 for falls. The overall Cronbach’s alpha coefficient for the scale was found to be 0.80.

### 2.5. Data Collection Method

The data were gathered from August 2023 to June 2024. To facilitate the “Paperless Hospital” initiative, the forms and scales were developed using Google Forms. The survey comprises three sections. The initial section provided an instructive paragraph outlining the study’s objective and scope, along with a checkbox for participants to indicate their approval for voluntary participation. The second section could not be passed without this approval. In the second section, there is a Personal Information Form for the nurses. In the “Intensive Care Unit” query within this section, only the respondents who selected a surgical intensive care unit were permitted to go to the third section. This measure was implemented to restrict the involvement of nurses not assigned to the surgical intensive care unit. The third section comprised the scales employed in this study. The data collection process concluded upon the participants’ completion of this section.

The researcher disseminated the Google Forms link to the nurses, who consented to participate in the study through social media. Participants were instructed to share the link with additional surgical intensive care nurses eligible for the survey. The sample size was achieved through the snowball sampling method. This process was implemented in six public hospitals within the province to access the sample. The data collection process required approximately 10 min.

### 2.6. Statistical Analysis

The data were analyzed using the Statistical Package for the Social Sciences–SPSS version 26.0 (IBM Corp, Armonk, NY, USA), a statistical software for the social sciences. Descriptive statistics involved calculating frequency and percentage distributions, while the evaluations concerning the scales included determining the average, standard deviation, and range of values. The suitability of the data for normal distribution was evaluated by analyzing skewness and kurtosis values. This study applied the Pearson correlation coefficient to examine the direction and strength of the relationship between variables that followed a normal distribution, while linear regression analysis was used to explore how these variables affected each other. Statistical significance was considered at *p* < 0.05. In addition, the Jamovi 2.5.3 program was used to test the moderator effects of age, gender, education level, and years of working in intensive care on the relationship between alarm fatigue and the tendency to make medical errors [23].

### 2.7. Ethical Principles of the Study

This study was approved by the non-interventional clinical research ethics committee of Çukurova University Faculty of Medicine (meeting number/decision number/date: 135/59/14 July 2023). Furthermore, verbal approval was secured from the appropriate institutional managers. Nurses were provided with detailed information about the study through Google Forms during the data collection process and were requested to select the voluntary participation checkbox. This method secured written consent from the nurses. The research adhered to the ethical principles outlined in the Helsinki Declaration established by the World Medical Association.

## 3. Results

The analysis of the descriptive characteristics of the participating nurses revealed a mean age of 31.69 ± 5.91 years. Additionally, 64.2% of the nurses were female, 64.2% were married, and 62.2% held undergraduate degrees. Furthermore, an analysis of the professional characteristics of the nurses revealed that 41.8% possessed 6–10 years of experience, 40.3% had worked in surgical intensive care units for 0–5 years, 71.6% were engaged in shift work (day-night), and 84.1% worked more than 40 h (Table 1).

Table 2 indicates that the mean total score of alarm fatigue among nurses in surgical intensive care units is 16.42 ± 5.47, reflecting a moderate level of alarm fatigue. The analysis revealed that this degree of alarm fatigue exerted a moderate adverse impact on nurses’ performance. The mean total score indicating nurses’ tendency for medical errors was found to be 180.57 ± 24.32, categorizing it as moderate. Table 2 presents the mean scores of nurses employed in surgical intensive care units, derived from the sub-dimensions of the tendency to make medical errors scale.

There is no relationship between age and the average alarm fatigue score (*p* = 0.311, r = 0.072). However, a relationship was found between age and the average tendency to make medical errors score (*p* = 0.001, r = −0.231). Specifically, as age increases, the tendency to make medical errors score decreases. A decrease in the tendency to make medical errors score leads to an increase in the likelihood of making medical errors. The average alarm fatigue and tendency to make medical errors scores showed statistically significant differences between male and female nurses (*p* < 0.001, respectively). The alarm fatigue scale scores were higher in male nurses, while the medical error scale scores were higher in female nurses. The alarm fatigue and tendency to make medical errors scores also showed statistically significant differences across educational levels (*p* < 0.001, respectively). As educational level increased, the average alarm fatigue score decreased, while the medical error scale score increased. The alarm fatigue and tendency to make medical errors scores showed statistically significant differences based on years of experience in intensive care (*p* < 0.001, respectively). As years of experience in intensive care increased, the average alarm fatigue score increased, while the medical error scale score decreased (Table 3).

This study revealed a negative moderate correlation (r = −0.381; *p* = 0.001) between the mean total score of alarm fatigue and the mean total score of the tendency to make medical errors among nurses in the surgical intensive care unit. A low negative correlation was observed between the mean score of alarm fatigue and the sub-dimension score of ‘medication and transfusion practices’ (r = −0.271; *p* = 0.001). A moderate negative correlation was identified between the mean score of alarm fatigue and the sub-dimension score of ‘hospital infections’ (r = −0.373; *p* = 0.001), as well as with the ‘patient monitoring and material safety’ sub-dimension score (r = −0.318; *p* = 0.001), the ‘communication’ sub-dimension score (r = −0.216; *p* = 0.002), and the ‘falls’ sub-dimension score (r = −0.424; *p* = 0.001). The findings suggest that as alarm fatigue increases, the score reflecting the tendency to make medical errors decreases; however, this decrease indicates an actual increase in the tendency to make medical errors (Table 4).

Analysis of Table 5 revealed a significant correlation between nurses’ alarm fatigue and their tendency to commit medical errors (R = −0.381, R^2^ = 0.145, F = 33.884, *p* = 0.001). The findings indicate that alarm fatigue among nurses in surgical intensive care units moderately elevated the likelihood of medical errors. Furthermore, alarm fatigue accounted for 14.5% of the tendency to commit medical errors (*p* = 0.001). The analysis of the *t*-test results for the significance of the regression coefficients revealed that nurses’ alarm fatigue (t = −41.329; *p* = 0.001) significantly influenced the tendency to commit medical errors. Specifically, a one-unit increase in alarm fatigue corresponded to an increase of 0.381 units in the tendency to make medical errors (*p* = 0.001) (Table 5).

It was observed that age moderates the relationship between alarm fatigue and nurses’ tendency to make medical errors (*p* = 0.002). The moderating effect of older age values was significant (*p* < 0.001), while the moderating effect of younger age values was not significant (*p* = 0.152). When the average age was increased by 1 standard deviation, a 2.695-unit increase in the tendency to make medical errors was observed. It was found that gender and education did not have an effect on the relationship between alarm fatigue and nurses’ tendency to make medical errors (*p* = 0.149, *p* = 0.446, respectively). Additionally, years of experience in the intensive care unit moderated the relationship between alarm fatigue and nurses’ tendency to make medical errors (*p* < 0.001). The moderating effect of longer years of ICU experience was significant (*p* < 0.001), while the moderating effect of shorter ICU experience was not significant (*p* = 0.152). When years of ICU experience increased by 1 standard deviation, a 3.559-unit increase in medical error scores was observed (Table 6).

## 4. Discussion

The results of this study show that surgical intensive care nurses experience a moderate level of alarm fatigue and this has a moderate effect on their performance (Table 2). A study in Turkey indicated that nurses experienced moderate fatigue [17], while research in China reported high fatigue levels [24], and a study in Korea found moderate fatigue attributed to alarms [25]. These findings indicate that the prevalence and impact of alarm fatigue may differ across countries. Factors such as health systems, technological infrastructure, workload, and personnel numbers, which vary by country, are believed to influence the levels of alarm fatigue [26].

This study found that nurses in surgical intensive care units exhibited a moderate tendency for medical errors (Table 2). Research indicates that nurses across various clinics exhibit a low tendency for committing medical errors [27,28,29]. A study involving 373 nurses employed in intensive care units across Turkey revealed a low tendency for medical errors among the nurses [17]. Studies in the literature indicate that the moderate tendency of nurses in surgical intensive care units to commit medical errors is likely linked to factors such as workload, alarm fatigue, and the unique dynamics of the unit. This situation indicates the necessity for the development of strategies tailored to surgical intensive care units.

In this study, when the relationship between alarm fatigue and the tendency to make medical errors was analyzed, a moderate negative correlation was found (Table 4). This finding indicates that as the alarm fatigue score increases, the tendency to make medical errors scale score decreases. However, according to the Medical Error Tendency Scale in Nursing, the tendency to make medical errors increases as the score obtained from this scale decreases [22]. This seeming contradiction is due to the scoring system of the scale, where lower scores indicate a higher tendency to make medical errors. As also stated in the study conducted by Gündoğan and Erdağı Oral, a lower score on this scale is associated with an increased tendency to make medical errors [17]. Furthermore, alarm fatigue among nurses accounted for 15% of the total variance in the tendency to commit medical errors (Table 5). The impact of alarm fatigue on medical errors is constrained yet remains a noteworthy consideration. Due to the absence of studies in the literature focusing on nurses in surgical intensive care units, this finding was compared with research conducted in general intensive care units [17]. Gündoğan and Erdağı Oral found a weak negative correlation between alarm fatigue levels and the mean total score of medical errors among nurses in all intensive care units. In the same study, fatigue level accounted for 6.9% of the total variance in the tendency to commit medical errors, and this relationship was statistically significant [17]. Although this study was similar to the literature, the variance was found to be high. The high variance is attributed to the number of devices in surgical intensive care units, reflecting the severity of patients’ conditions and the demands of postoperative care, which necessitate intensive medical intervention and monitoring.

This study revealed a weak negative correlation between alarm fatigue and its sub-dimensions, including medication and transfusion practices, as well as communication. Alarm fatigue exhibited a moderate negative correlation with sub-dimensions, including nosocomial infections, patient monitoring, material safety, and falls. Increased alert fatigue correlates with a higher tendency for medical errors (Table 4). A related study indicated that heightened alarm fatigue corresponded with elevated scores in the sub-dimension of the tendency to commit medical errors scale [17]. These findings align with the current literature. The exposure of nurses to alarm sounds may hinder their ability to concentrate on treatment, care, and follow-up practices, thereby increasing the likelihood of medical errors. Another study supports this interpretation, indicating that 44% of nurses in intensive care units experienced interruptions in sustainable treatment and care due to alarms. Additionally, the study indicates that each interruption during a drug preparation process raises the probability of errors by 25% due to the necessity of restarting the process [30]. Other studies in the literature indicate that alarm fatigue impacts working discipline by inducing distraction, cognitive overload, and difficulties in concentration among nurses, which directly correlates with a higher tendency for medical errors [17,25,30,31,32,33].

This study found that a one-unit increase in alarm fatigue among nurses in the surgical intensive care unit was associated with a 0.381 times increase in the tendency for medical errors (Table 5). While studies often reference the impact of alarm fatigue on medical errors, they typically lack detailed information regarding the strength and correlation of this effect. A study across all intensive care units indicated that a one-unit increase in alarm fatigue correlates with a 0.263-unit increase in the likelihood of nurses committing medical errors [17]. The data presented in this article align with the study; however, the effects observed in the study were more pronounced. The increased utilization of medical devices for monitoring postoperative patients in surgical intensive care units is believed to be the cause.

## 5. Limitations and Strengths

This study is one of the original studies examining the relationship between alarm fatigue and the tendency to make medical errors, and it makes an important contribution to the literature. In this study, scales with proven validity and reliability were used, which increases the scientific validity of the data obtained. In addition, this study provides clinically valuable findings that may help healthcare professionals better understand the relationship between alert fatigue and the propensity to make medical errors, which is a critical issue for patient safety. However, this study was conducted in hospitals in one province of a particular country. Therefore, there are limitations in generalizing the results to all countries or a large region. In addition, since the data were collected through online forms based on participants’ self-reports, the response process could not be controlled. This suggests that factors such as social desirability bias may have played a role in the accuracy of the responses.

## 6. Future Directions

The findings of this study contribute to the relationship between fatigue and error rates in medical personnel. Future studies conducted in different health institutions and with larger samples will increase the generalizability of the results. In addition, studies that include factors such as demographic characteristics, working hours, workload, shift patterns, and psychological resilience may provide a more comprehensive view of the subject. In addition to quantitative data, in-depth analyses of the experiences of healthcare workers through qualitative methods are also recommended. Finally, experimental studies evaluating the effectiveness of intervention programs for reducing fatigue will contribute to practical applications.

## 7. Clinical Applications

Alarm systems are critical in surgical intensive care units, and it is important to monitor the levels of alarm fatigue among nurses. Institutions must develop protocols and practices to improve patient safety and reduce medical errors in these units. A customized approach to managing alarms at each institution is essential for addressing alarm fatigue and improving patient safety. Nurses should be trained in alarm management practices to raise their awareness of alarm-related concerns. Institutions can improve the alarm management skills of intensive care nurses through training programs aimed at reducing alarm fatigue. Conducting research to assess the psychological and physiological impacts on nurses is vital for ensuring the safety of healthcare workers.

## 8. Conclusions

This study aimed to evaluate the effect of alarm fatigue on the likelihood of medical errors among nurses in surgical intensive care units. The study indicated that alarm fatigue and the tendency for medical errors among surgical ICU nurses were at a moderate level. A moderate negative correlation was found between alarm fatigue and the likelihood of making medical errors. This suggests that as alarm fatigue increases, the score indicating the tendency to make medical errors decreases; however, this decline in the score implies a higher tendency to commit medical errors. Additionally, each increase in alert fatigue was linked to a 0.381-unit rise in the likelihood of medical errors. In conclusion, alarm fatigue among nurses in surgical intensive care units negatively affects patient safety.

## Figures and Tables

**Table 1:** Descriptive characteristics of nurses.

	Mean ± SD	(Min–Max)
Age (Years)	31.69 ± 5.91	(23–49)
	**Number (n)**	**Percentage (%)**
Gender		
Female	129	64.2
Male	72	35.8
Marital status		
Married	129	64.2
Single	72	35.8
Education level		
Health vocational high school	16	8.0
Associate degree	32	15.9
Undergraduate	125	62.2
Postgraduate	28	13.9
Working time in the profession		
0–5 years	69	34.3
6–10 years	84	41.8
11–15 years	24	11.9
16 years and over	24	12.0
Intensive care unit worked in		
General surgery intensive care	40	19.9
Brain surgery intensive care	36	17.9
Coronary intensive care	28	13.9
Cardiovascular intensive care	28	13.9
Surgical intensive care	45	22.5
Pediatric surgical intensive care	24	11.9
Working time in intensive care unit		
0–5 years	81	40.3
6–10 years	72	35.8
11–15 years	36	17.9
16 years and over	12	6.0
Working type		
Shift	144	71.6
Daytime	57	28.4
Weekly working hours		
40 h	32	15.9
40 h and more	169	84.1
Total	279	100

**Table 2:** Mean scores of nurses related to alarm fatigue and medical error tendency scale subscales.

Scales and Sub-Dimensions	Number of Items	Min	Max	Mean ± SD
Alarm Fatigue Scale	9	7.00	27.00	16.42 ± 5.47
Medical Error Tendency Scale	49	126.00	230.00	180.57 ± 24.32
Medication and transfusion practices	18	44.00	121.00	72.67 ± 11.45
Hospital infections	12	24.00	45.00	33.85 ± 5.93
Patient monitoring and material safety	9	24.00	45.00	33.99 ± 6.20
Communication	5	15.00	25.00	20.43 ± 2.66
Falls	5	14.00	25.00	19.62 ± 3.33

**Table 3:** Comparison of the mean scores of alarm fatigue and tendency to make medical errors according to the demographic and occupational characteristics of nurses.

	Alarm Fatigue Scale Mean ± SD	Medical Error Tendency Scale Mean ± SD
Gender		
Female	14.14 ± 5.03	192.37 ± 22.01
Male	20.50 ± 3.50	167.44 ± 25.02
*p* value	<0.001	<0.001
Marital status		
Married	14.92 ± 5.40	190.82 ± 23.48
Single	19.11 ± 4.50	170.22 ± 25.16
*p* value	<0.001	<0.001
Education level		
Health vocational high school	21.50 ± 3.05	154.25 ± 11.69
Associate degree	21.00 ± 3.13	175.62 ± 32.17
Undergraduate	14.08 ± 5.10	192.83 ± 22.20
Postgraduate	18.71 ± 3.83	167.14 ± 14.70
*p* value	<0.001	<0.001
Working time in the profession		
0–5 years	14.39 ± 5.14	193.62 ± 18.22
6–10 years	16.76 ± 5.70	185.19 ± 29.54
11–15 years	19.66 ± 4.47	165.50 ± 14.74
16–20 years	16.00 ± 4.06	178.75 ± 16.66
*p* value	<0.001	<0.001
Intensive care unit worked in		
General surgery intensive care	19.00 ± 4.83	173.80 ± 23.71
Brain surgery intensive care	18.00 ± 3.85	180.55 ± 29.38
Coronary intensive care	15.71 ± 5.43	176.14 ± 30.31
Cardiovascular intensive care	17.71 ± 5.03	179.00 ± 24.92
Surgical intensive care	12.55 ± 5.55	193.60 ± 18.12
Pediatric surgical intensive care	16.33 ± 5.19	198.50 ± 21.69
*p* value	<0.001	<0.001
Working time in intensive care unit		
0–5 years	14.67 ± 5.49	189.67 ± 19.90
6–10 years	16.16 ± 5.40	191.00 ± 27.89
11–15 years	19.66 ± 4.27	165.33 ± 8.47
16 years and over	20.00 ± 2.55	150.33 ± 19.07
*p* value	<0.001	<0.001
Working type		
Shift	16.50 ± 5.43	184.41 ± 24.58
Daytime	16.22 ± 5.60	180.98 ± 29.34
*p* value	0.752	0.400
Weekly working hours		
40 h	16.75 ± 6.02	167.50 ± 21.18
40 h and more	16.36 ± 5.37	186.46 ± 25.77
*p* value	0.713	<0.001

**Table 4:** The relationship between alarm fatigue and the tendency to medical errors of nurses working in surgical intensive care units.

Medical Error Tendency Scale and Its Sub-Dimensions		Alarm Fatigue Scale
Medical Error Tendency Scale	r ^a^	−0.381 *
	*p* value	<0.001
Medication and transfusion practices	r ^a^	−0.271 *
	*p* value	<0.001
Hospital infections	r ^a^	−0.373 *
	*p* value	<0.001
Patient monitoring and material safety	r ^a^	−0.318 *
	*p* value	<0.001
Communication	r ^a^	−0.216 *
	*p* value	<0.002
Falls	r ^a^	−0.424 *
	*p* value	<0.001

^a^: Pearson correlation, * *p* < 0.001.

**Table 5:** The effect of alarm fatigue on the tendency to make medical errors in nurses working in surgical intensive care units.

Variables		B	Standard Error	Beta	t	*p*
Tendency to Make Medical Errors	Constant	208.437	5.043		41.329	0.001 *
	Alarm Fatigue	−1.696	0.291	−0.381	−5.821	0.001 *
	R = −0.381, R^2^ = 0.145, F = 33.884, *p* = 0.001 *					

Linear regression analysis was performed. * *p* < 0.001.

**Table 6:** The moderator effect of nurses’ demographic and occupational characteristics on the relationship between alarm fatigue and the tendency to make medical errors.

	Estimate	SE	95% Confidence		*p*
			Lower	Upper	
The Moderating Effect of Age					
Alarm Fatigue Scale	−1.680	0.2970	−2.262	−1.0979	<0.001
Age	−0.727	0.2745	−1.265	−0.1887	0.008
Alarm Fatigue Scale × Age	−0.172	0.0565	−0.283	−0.0614	0.002
Average	−1.680	0.306	−2.28	−1.081	<0.001
Low (−1 SD)	−0.665	0.465	−1.58	0.246	0.152
High (+1 SD)	−2.695	0.445	−3.57	−1.824	<0.001
The Moderating Effect of Age and Gender					
Alarm Fatigue Scale	−0.963	0.293	−1.54	−0.390	<0.001
Gender	−16.874	3.330	−23.40	−10.348	<0.001
Alarm Fatigue Scale × Gender	−1.073	0.744	−2.53	0.385	0.149
The Moderating Effect of Education					
Alarm Fatigue Scale	−1.695	0.311	−2.305	−1.08	<0.001
Education	1.401	2.209	−2.929	5.73	0.526
Alarm Fatigue Scale × Education	0.331	0.435	−0.521	1.18	0.446
The Moderating Effect of Years of Experience in Intensive Care					
Alarm Fatigue Scale	−1.65	0.274	−2.19	−1.12	<0.001
Intensive care unit worked in	−5.65	1.648	−8.87	−2.42	<0.001
Alarm Fatigue Scale × Intensive care unit worked in	−2.12	0.343	−2.79	−1.44	<0.001
Average	−1.654	0.305	−2.252	−1.06	<0.001
Low (−1 SD)	0.251	0.421	−0.574	1.08	0.550
High (+1 SD)	−3.559	0.466	−4.473	−2.65	<0.001

SE: Standard error.

## Data Availability

The data that support the findings of this study are available on request from the corresponding author. The data are not publicly available due to privacy or ethical restrictions.
